# Fetal Akinesia/Hypokinesia and Arthrogryposis of Neuromuscular Origin: Etiologic Groups, Genetics, and Phenotypic Spectrum

**DOI:** 10.1002/acn3.70088

**Published:** 2025-05-29

**Authors:** Florencia Pérez‐Vidarte, Berta Estévez‐Arias, Leslie Matalonga, Delia Yubero, Anna Codina, Carlos Ortez, Julita Medina, Lidia DeSena DeCabo, Laura Carrera‐García, Jesica Expósito‐Escudero, Cristina Jou, Eduardo F. Tizzano, Andres Nascimento, Daniel Natera‐de Benito

**Affiliations:** ^1^ Neuromuscular Unit, Department of Neurology Hospital Sant Joan de Déu Barcelona Spain; ^2^ Laboratory of Neurogenetics and Molecular Medicine Center for Genomic Sciences in Medicine, Institut de Recerca Sant Joan de Déu Barcelona Spain; ^3^ Centro Nacional de Análisis Genómico (CNAG) Barcelona Spain; ^4^ Universitat de Barcelona (UB) Barcelona Spain; ^5^ Center for Biomedical Research Network on Rare Diseases (CIBERER), ISCIII Spain; ^6^ Department of Genetic and Molecular Medicine Hospital Sant Joan de Déu Barcelona Spain; ^7^ Applied Research in Neuromuscular Diseases Institut de Recerca Sant Joan de Déu Barcelona Spain; ^8^ Department of Pathology Hospital Sant Joan de Déu Barcelona Spain; ^9^ Rehabilitation and Physical Unit Department Hospital Sant Joan de Deu Barcelona Spain; ^10^ Department of Pediatric Orthopaedic Surgery Hospital Sant Joan de Deu Barcelona Spain; ^11^ Medicine Genetics Group Vall d'Hebron Institut de Recerca (VHIR) Barcelona Spain

**Keywords:** Amyoplasia, *CHRNG*, *DYNC1H1*, myopathy, neuropathy, *PIEZO2*, pterygium multiple, *TTN*, *ZC4H2*

## Abstract

**Objective:**

To provide a comprehensive clinical and genetic characterization of individuals with arthrogryposis multiplex congenita (AMC), focusing on the distribution of genetic etiologies across the neuromuscular spectrum and comparing myogenic and neurogenic subtypes.

**Methods:**

A total of 105 individuals with AMC were clinically and genetically evaluated in a single‐center study. Participants were stratified based on the primary site of involvement, and further classification was performed for neuromuscular cases into neurogenic and myogenic subtypes. Genetic diagnoses were made through using a range of next‐generation sequencing techniques, including exome sequencing (42 individuals), gene panel testing (40 individuals), genome sequencing (24 individuals), and targeted‐gene testing in selected cases. In most individuals who underwent genome sequencing, this was preceded by exome or gene panel testing.

**Results:**

Of the 105 individuals, 4 were classified as Amyoplasia and 1 as FARAD. Among the remaining 100 cases, 81 (81%) presented with neuromuscular AMC, with defects involving motor neurons/peripheral nerves (52%, 42/81), neuromuscular junctions (7%, 6/81), and skeletal muscle (41%, 33/81). A genetic diagnosis was achieved in 55% (55/100) of the entire cohort and in 58% (47/81) of individuals with neuromuscular AMC. The most frequently implicated genes were *TTN* (16%, 9/55), *CHRNG* (10.9%, 6/55), *PIEZO2* (9.1%, 5/55), *ZC4H2* (9.1%, 5/55), *DYNC1H1* (7.2%, 4/55), *MYH3* (5.4%, 3/55), and *RYR1* (5.4%, 3/55). Diagnostic yield varied significantly between subgroups, with 84.6% (33/39) of myogenic AMC cases genetically resolved, compared to 33.3% (14/42) of neurogenic cases. *TTN* was the most common gene in myogenic AMC, while *ZC4H2* and *DYNC1H1* were predominant in neurogenic AMC.

**Interpretation:**

This study provides a detailed phenotypic and genetic characterization of neuromuscular AMC, highlighting the most frequently affected genes and their associated phenotypes. The findings underscore the challenges in diagnosing a significant proportion of cases, especially within the neurogenic subgroup, and emphasize the importance of integrating detailed phenotypic data with genetic analysis to enhance diagnosis, prognosis, and management of family expectations.

**Abbreviations**
AMC: arthrogryposis multiplex congenita
DA: distal arthrogryposis
ENMG: electroneuromyography
FADS: fetal akinesia deformation sequence
LCCS: lethal congenital contracture syndrome
MPS: multiple pterygium syndrome
NCS: nerve conduction studies
NGS: next‐generation sequencing
SD: standard deviations

## 1 Introduction

Fetal akinesia/hypokinesia is the common mechanism leading to a range of clinical presentations characterized by joint contractures due to limited movement during the prenatal period. Arthrogryposis multiplex congenita (AMC) is an umbrella term that encompasses a wide range of disorders with congenital joint contractures, including more specific clinical entities such as distal arthrogryposis (DA), fetal akinesia deformation sequence (FADS), lethal congenital contracture syndrome (LCCS), and multiple pterygium syndrome (MPS), among others [1, 2, 3]. Some individuals with these conditions exhibit overlapping features that are also a direct result of reduced prenatal mobility, such as fetal hydrops, lung hypoplasia, and dysmorphic features.

The underlying defects causing fetal akinesia/hypokinesia can be classified into five major groups, which do not necessarily account for an equal proportion of cases: genetic alterations, congenital infections, extrinsic causes (such as oligohydramnios, amniotic bands, or anatomical abnormalities of the uterus), maternal immune diseases like myasthenia gravis [4], and Amyoplasia. To date, more than 400 genes have been associated with AMC [4, 5]. Several previous cohort studies have observed that the genes most commonly affected in individuals with AMC are those involved in neuromuscular function, specifically genes encoding key components of motor neurons, peripheral nerves, neuromuscular junctions, or skeletal muscle [3, 4, 5, 6, 7, 8]. However, Amyoplasia, which predominantly affects the muscles, is considered a distinct entity characterized by limb involvement and is thought to have a vascular or developmental etiology, rather than being a primary genetic neuromuscular disorder [9].

To enhance clarity in the classification of neuromuscular AMC, we distinguish two main groups based on the primary site of dysfunction: neurogenic and myogenic arthrogryposis. Neurogenic AMC arises from defects in the motor neuron or peripheral nerve, leading to denervation, impaired muscle activation, and secondary joint contractures. Myogenic AMC results from primary abnormalities in the muscle or neuromuscular junction, particularly affecting excitation‐contraction coupling and leading to congenital contractures. This distinction is relevant for improving genotype–phenotype correlation and refining diagnostic approaches.

Despite the increasing rate of molecular diagnosis in individuals with arthrogryposis, facilitated by the accessibility of next‐generation sequencing (NGS) methods, the genetic cause remains unknown in approximately 30%–50% of cases [4, 5, 6]. The distribution of genetic subtypes of arthrogryposis within a neuromuscular unit has not been previously addressed. A better understanding of the distribution of these subtypes, as well as deeper insights into the relationship between phenotypes and genotypes, would be highly beneficial for corroborating genetic findings, which are often challenging to interpret.

In this single‐center study, we clinically and genetically characterized a historical cohort of 105 individuals with AMC. The individuals were stratified based on the primary site of involvement, with those presenting neuromuscular AMC further classified into neurogenic and myogenic subtypes. Our objectives were to characterize the phenotypic and genetic landscape of these groups, identify the most frequently implicated genes, define their associated clinical features, and assess the characteristics of individuals who remain without a genetic diagnosis.

## 2 Methods

### 2.1 Study Design, Inclusion Criteria, and Data Collection

This retrospective cross‐sectional study was conducted at a national and European reference center for pediatric neuromuscular disorders (Hospital Sant Joan de Déu, Barcelona). Data were collected from all individuals diagnosed with AMC, DA, MPS between January 2010 and December 2024. The study adhered to the guidelines set forth by the Clinical Ethics Committee of the hospital (Ethics Committee Reference PIC 147‐23).

Phenotypic and genetic data were collected from medical records, interviews with individuals with arthrogryposis and their families, and physical examinations. Detailed reviews of electromyography (EMG) and nerve conduction studies (NCS), as well as muscle biopsies, were conducted specifically for this study.

The demographic information collected included age, sex, and ethnic origin. Natural history data encompassed the history of motor developmental milestones, current motor function level, and age at genetic diagnosis. Additional relevant medical history was documented, including details on muscle weakness and its distribution, respiratory function, scoliosis, and bulbar involvement (e.g., gastrostomy insertion). Cognitive function was also gathered from the medical records, although neuropsychological assessments were not systematically conducted for all individuals.

### 2.2 Genetic and Genomic Analyses

Genetic analyses were conducted based on the clinical, neurophysiological, and histological phenotypes, utilizing various NGS techniques, depending on their availability and the specific phenotype. These techniques ranged from customized panels targeting selected genes to genome sequencing, with exome sequencing being the most frequently employed performed in 42 individuals, followed by gene panel testing (in 40 individuals), and genome sequencing (in 24 individuals). In most individuals who underwent genome sequencing, prior testing with exome sequencing or gene panels had already been performed. The most commonly used methods included the Custom Comprehensive panel 17 Mb (Agilent Technologies, USA), the Nextera Rapid Capture (Illumina, USA), and the TruSight One Sequencing Panel (Illumina, USA). Gene panels evolved over time, incorporating newly discovered genes and adapting to the most current knowledge. Informed consent was obtained from parents in every case.

Targeted‐gene testing was mainly done in earlier years for individuals with clinical suspicion of specific genetic causes. It was performed in 12 individuals, leading to a diagnosis in 3 with *CHRNG*‐related AMC, 3 with *RYR1*‐related congenital myopathy, 2 with *PIEZO2* variants, and 1 with *TRPV4*. Array CGH was performed in 2 individuals, but no diagnosis was found. Additionally, in 4 individuals, the genetic diagnosis was established through segregation studies after a pathogenic variant had been identified in a similarly affected family member.

### 2.3 Etiological Stratification

Eight etiological subgroups were defined based on obstetric history, clinical examination, and complementary tests, with consideration of both the underlying etiology and the pathophysiological processes, particularly the structures involved. These groups were selected based on the various known etiologies and adapted from the disease classification framework proposed by Le Tanno et al. [4]. Classification as neurogenic or myogenic arthrogryposis was determined through a combination of neurophysiological and clinical findings, with muscle pathology providing additional information in cases where it was available. The involvement of specific structures, such as muscles, nerves, or joints, was crucial in determining the pathogenesis of each subgroup. The eight etiological groups were: (i) Maternal causes (including space limitations and maternal illness or exposures), (ii) Amyoplasia, (iii) Central nervous system (CNS) defects without peripheral nerve defects, (iv) Peripheral nerve defects, (v) Neuromuscular junction defects, (vi) Myopathies (including tension‐sensing genes), (vii) Metabolic disorders, and (viii) Connective tissue and skeletal dysplasia. Amyoplasia was defined based on the clinical diagnosis established by Hall et al. [9]. Individuals with characteristics compatible with two different etiological groups were classified into the group considered most predominant. Those who did not exhibit clear features to be classified into any of the proposed groups were categorized under “unknown mechanism(s)” arthrogryposis.

### 2.4 Statistical Analysis

Statistical analyses were performed using the Statistical Package for the Social Sciences, version 24.0 (IBM SPSS Statistics 24.0, USA). Descriptive statistics were used to summarize the demographic and clinical characteristics of the cohort. Data are presented as means ± standard deviations (SD) for normally distributed variables or as medians and ranges for skewed variables.

Comparisons between groups (e.g., patients with different genetic diagnoses or those with and without a genetic diagnosis) were conducted using chi‐square tests for categorical variables. The significance level was set at *p* < 0.05.

## 3 Results

### 3.1 Demographic and Clinical Characteristics of the Whole Cohort

**FIGURE 1:**
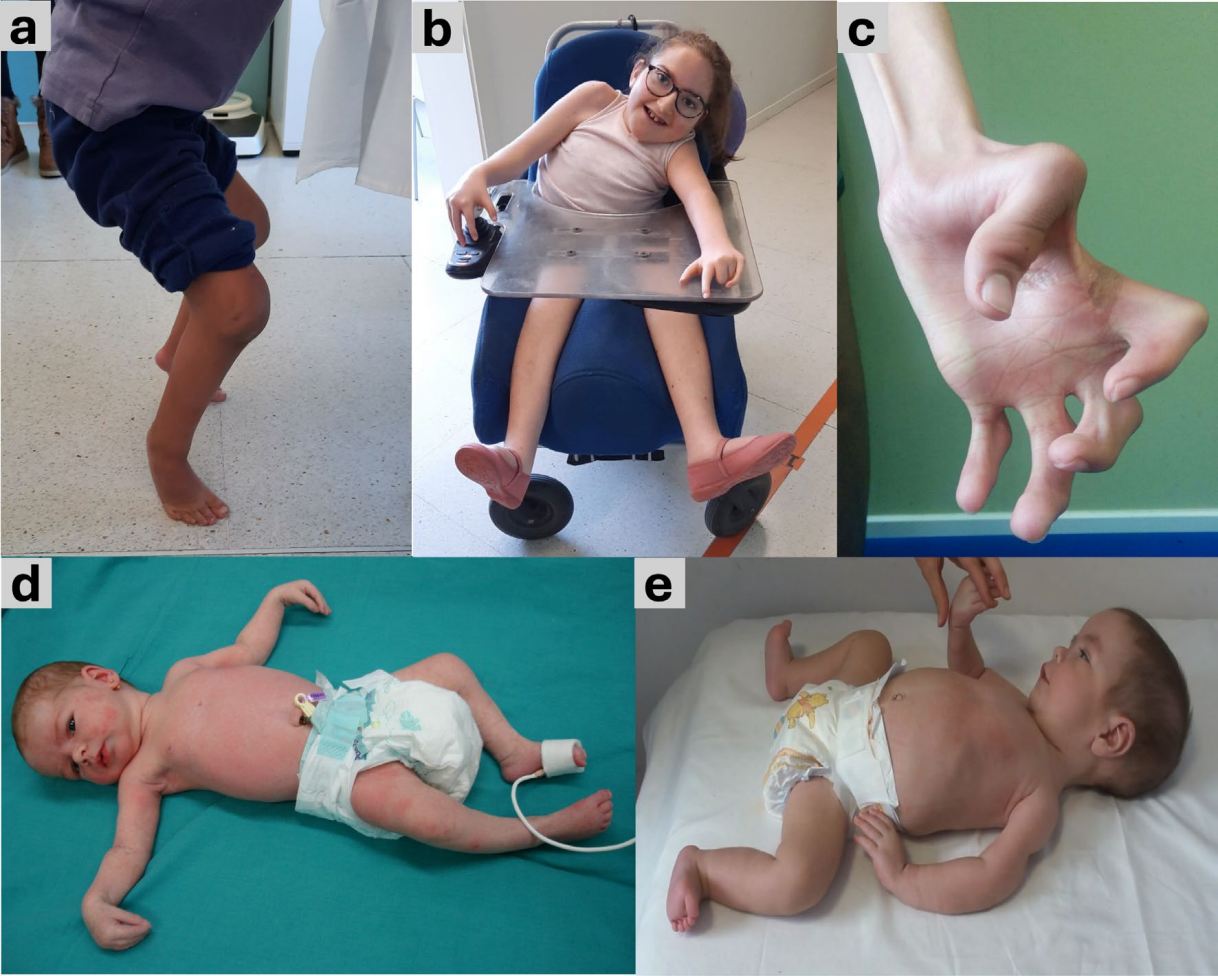
Clinical images of individuals with AMC. (a) A female with AMC caused by a monoallelic *PIEZO2* variant, showing retractions of the hip flexors, knees, and Achilles tendons, preventing autonomous bipedal stance; (b) A 7‐year‐old female with *ZC4H2*‐related AMC, note the inability to flex the knees; (c) Hand of a 14‐year‐old male with *CHRNG*‐related AMC, associated with multiple pterygium syndrome; (d) A 3‐month‐old female with severe *LAMA2*‐related congenital muscular dystrophy, note the congenital contractures in the hands and knees; (e) A male infant with *TTN*‐related congenital myopathy, presenting with joint contractures and an open‐book posture due to muscle weakness.

Our cohort of individuals with AMC followed up at our neuromuscular unit totaled 105 index cases (58 males and 47 females) from 101 unrelated families. Representative images of some individuals are shown in Figure 1. Clinical and genetic data are summarized in Tables 1, 2, 3. Electroneuromyography (ENMG) was performed in 96% (101/105) of individuals (101/105), reflecting its role as the primary diagnostic tool in our Neuromuscular Unit to identify whether AMC is of neurogenic or myogenic origin. Muscle biopsy was carried out in 36% (38/105), typically in cases with ENMG findings suggestive of a myogenic pattern. No muscle biopsies were performed without prior ENMG testing. Whole‐body muscle MRI was conducted in 17 individuals (16%).

**TABLE 1:** Clinical and genetic information of all genetically diagnosed individuals with myogenic AMC of our cohort.

Ind	Fam	Sex	Age last seen/death (year)	Etiological group	Gene (pattern of inheritance)	Variant(s) ACMG evidence of pathogenicity	Age at independent gait (year)	Respiratory Support (age; year)	Scoliosis (Surgery: Yes/No; age at surgery (year))	Cognitive involvement
1	1	M	22	Myogenic (including NMJ and tension sensing genes)	*TTN* (AR)	**c.35876‐2A>G** (P (PVS1, PS4, PM2)) **c.50248 + 1G>C** (LP (PVS1, PM2, PM3))	4	No	No	No
2	2	F	9	Myogenic (including NMJ and tension sensing genes)	*TTN* (AR)	c.33055del; p.Glu11019Serfs*21 (P (PVS1, PM2, PM3)) c.38737G>T; p.Glu12913* (P (PVS1, PS4, PM2))	Not achieved	NNIV (6 year)	Yes (no surgery)	No
3	3	M	30	Myogenic (including NMJ and tension sensing genes)	*TTN* (AR)	c.38737G>T; p.Glu12913* (P (PVS1, PS4, PM2)) c.67495C>T; p.Arg22499* (P (PVS1, PS4, PM2))	Not achieved	No	Yes (surgery at 14)	No
4	4	M	29	Myogenic (including NMJ and tension sensing genes)	*TTN* (AR)	c.38661_38665del; p.Lys12887Asnfs*6 (P (PVS1, PM2, PM3)) c.103531A>T; p.Lys34511* (P (PVS1, PM2, PM3))	4	No	Yes (no surgery)	No
5	5	F	17	Myogenic (including NMJ and tension sensing genes)	*TTN* (AR)	c.38661_38665del; p.Lys12887Asnfs*6 (P (PVS1, PM2, PM3)) **c.17741‐1G>A** (LP (PVS1, PM2, PM3))	Not achieved	No	Yes (no surgery)	No
6	6	M	12	Myogenic (including NMJ and tension sensing genes)	*TTN* (AR)	c.38661_38665del; p.Lys12887Asnfs*6 (P (PVS1, PM2, PM3)) *Maternal isodisomy*	Not achieved	No	Yes (surgery at 11)	ID and ASD
7	7	M	9	Myogenic (including NMJ and tension sensing genes)	*TTN* (AR)	c.38661_38665del; p.Lys12887Asnfs*6 (P (PVS1, PM2, PM3)) **c.59626G>A; p.Asp19876Asn** (VUS (PP3, PM2, PM3))	Not achieved	No	Yes (no surgery)	No
8	8	F	34	Myogenic (including NMJ and tension sensing genes)	*TTN* (AR)	c.35756del; p.Pro11919Leufs*51 (P (PVS1, PM2, PM3)) c.79663G>T; p.Glu26555* (P (PVS1, PM2, PM3))	2	No	No	No
9	8	M	33	Myogenic (including NMJ and tension sensing genes)	*TTN* (AR)	c.35756del; p.Pro11919Leufs*51 (P (PVS1, PM2, PM3)) c.79663G>T; p.Glu26555* (P (PVS1, PM2, PM3))	1,5	No	No	No
10	9	F	7	Myogenic (including NMJ and tension sensing genes)	*CHRNG* (AR)	**c.299 T>G; p.Leu100Arg** (VUS (PP3, PM2, PM3)) **c.299 T>G; p.Leu100Arg** (VUS (PP3, PM2, PM3))	1,2	No	No	No
11	10	F	18	Myogenic (including NMJ and tension sensing genes)	*CHRNG* (AR)	**c.715C>T; p.Arg239Cys** (P (PS3, PP1, PP3, PM2, PM3)) **c.715C>T; p.Arg239Cys** (P (PS3, PP1, PP3, PM2, PM3))	1,2	No	Yes (no surgery)	No
12	11	F	27	Myogenic (including NMJ and tension sensing genes)	*CHRNG* (AR)	c.753_754del; p.Val253Alafs*44 (P (PVS1, PM2, PM3)) c.753_754del; p.Val253Alafs*44 (P (PVS1, PM2, PM3))	1,2	No	Yes (no surgery)	No
13	12	M	18	Myogenic (including NMJ and tension sensing genes)	*CHRNG* (AR)	c.459dup; p.Val154Serfs*24 (P (PVS1, PM2, PM3)) c.459dup; p.Val154Serfs*24 (P (PVS1, PM2, PM3))	1	No	Yes (no surgery)	No
14	12	M	22	Myogenic (including NMJ and tension sensing genes)	*CHRNG* (AR)	c.459dup; p.Val154Serfs*24 (P (PVS1, PM2, PM3)) c.459dup; p.Val154Serfs*24 (P (PVS1, PM2, PM3))	1	No	Yes (no surgery)	No
15	13	M	9	Myogenic (including NMJ and tension sensing genes)	*CHRNG* (AR)	c.753_754delCT; p.Val253Alafs*44 (P (PVS1, PM2, PM3)) **c.257G>A; p.Arg86His** (LP (PP3, PM2, PM5))	2	No	No	ASD
16	14	M	17	Myogenic (including NMJ and tension sensing genes)	*PIEZO2* (AD)	**c.8396G>A; p.Arg2799His** (P (PS2, PP2, PP3, PM2, PM5))	1,5	No	Yes (no surgery)	No
17	15	F	7	Myogenic (including NMJ and tension sensing genes)	*PIEZO2* (AD)	**c.8520_8522del; p.Glu2840del** (P (PS3, PS4, PM1, PM2, PM4, PM6))	2	No	No	No
18	16	F	9	Myogenic (including NMJ and tension sensing genes)	*PIEZO2* (AR)	c.2004delG; p.Glu668Aspfs*5 (P (PVS1, PM2, PM3)) c.2004delG; p.Glu668Aspfs*5 (P (PVS1, PM2, PM3))	Not achieved	No	Yes (no surgery)	No
19	17	M	19	Myogenic (including NMJ and tension sensing genes)	*PIEZO2* (AR)	c.1084C>T, p.Gln362* (P (PVS1, PM2, PM3)) c.5227C>T, p.Arg1743* (P (PVS1, PS3, PM2, PM3))	5	No	Yes (surgery at 19)	No
20	18	M	18	Myogenic (including NMJ and tension sensing genes)	*PIEZO2* (AR)	c.8069C>A, p.Ser2690* (LP (PVS1, PM2)) **c.7779 + 1G>A** (VUS (PVS1, PM2))	Not achieved	No	Yes (no surgery)	No
21	19	M	9	Myogenic (including NMJ and tension sensing genes)	*RYR1* (AD)	**c.14819C>T; p.Ala4940Val** (LP (PP2, PP3, PM1, PM2, PM5, PM6))	Not achieved	NNIV (8 m)	Yes (surgery at 3)	No
22	20	M	23	Myogenic (including NMJ and tension sensing genes)	*RYR1* (AD)	**c.13732 T>G; p.Leu4578Val** (LP (PP2, PP3, PM1, PM2, PM6))	Not achieved	No	Yes (surgery at 16)	No
23	21	F	23	Myogenic (including NMJ and tension sensing genes)	*RYR1* (AR)	**c.13691G>A; p.Arg4564Gln** (P (PP2, PP3, PM1, PM2, PM3, PM5)) **c.13892A>G; p.Tyr4631Cys** (LP (PP2, PP3, PM1, PM2, PM3, PM5))	Not achieved	NNIV (12 y)	Yes (surgery at 12)	No
24	22	F	0,1 (death)	Myogenic (including NMJ and tension sensing genes)	*NEB* (AR)	c.22648_22649insA; p.Ser7550Tyrfs*24 (LP (PVS1, PM2)) **c.1782 + 4A>T** (VUS (PP3, PM2))	< 1 year at last visit	Death from respiratory failure	< 1 year at last visit	< 1 year at last visit
25	22	M	0,1 (death)	Myogenic (including NMJ and tension sensing genes)	*NEB* (AR)	c.22648_22649insA; p.Ser7550Tyrfs*24 (LP (PVS1, PM2)) **c.1782 + 4A>T** (VUS (PM2, PP3))	< 1 year at last visit	Death from respiratory failure	< 1 year at last visit	< 1 year at last visit
26	23	M	14	Myogenic (including NMJ and tension sensing genes)	*COL6A1* (AR)	c.817 A>T; p. Lys273* (P (PVS1, PM2, PM3)) c.817 A>T; p. Lys273* (P (PVS1, PM2, PM3))	Not achieved	NNIV (8 year)	Yes (no surgery)	No
27	23	M	11	Myogenic (including NMJ and tension sensing genes)	*COL6A1* (AR)	c.817 A>T; p. Lys273* (P (PVS1, PM2, PM3)) c.817 A>T; p. Lys273* (P (PVS1, PM2, PM3))	Not achieved	NNIV (7 year)	Yes (no surgery)	No
28	24	F	12	Myogenic (including NMJ and tension sensing genes)	*MYH3* (AD)	**c.737G>C;p.Gly246Ala** (LP (PP3, PM2, PM6))	1,3	No	No	No
29	25	M	14	Myogenic (including NMJ and tension sensing genes)	*MYH3* (AD)	**c.1141 + 1G>A** (LP (PVS1, PM2))	0,9	No	No	No
30	26	F	10	Myogenic (including NMJ and tension sensing genes) Distal arthrogryposis	*MYH3* (AD)	**c.2591 T>C; p.Leu864Pro** (VUS (PP3, PM1, PM2))	1,1	No	Yes (no surgery)	No
31	27	F	0,5	Myogenic (including NMJ and tension sensing genes)	*ACTA1* (AR)	c.541delG; p.Asp181Thrfs*11 (P (PVS1, PM2, PM3)) c.541delG; p.Asp181Thrfs*11 (P (PVS1, PM2, PM3))	< 1 year at last visit	TRAC‐NIV (< 1 year)	< 1 year at last visit	< 1 year at last visit
32	28	F	13	Myogenic (including NMJ and tension sensing genes)	*TPM2* (AD)	**c.397C>T; p.Arg133Trp** (P (PS3, PS2, PP2, PP3, PM1, PM2, PM5))	1,4	No	No	No
33	29	F	14	Myogenic (including NMJ and tension sensing genes)	*LAMA2* (AR)	c.442dup; p.Arg148Profs*12 (P (PVS1, PM2, PM3)) c.1854_1861dup; p.Leu621Hisfs*7 (P (PVS1, PM2, PM3))	Not achieved	NNIV (9 year)	Yes (surgery at 14)	No

*Note:* Variant classification was done based on the American College of Medical Genetics and Genomics (ACMG) guidelines [19], using Franklin (last access November 2024). Evidence of pathogenic and/or benign impact of each variant is indicated. All variants are annotated in hg19/GRCh37 and nucleotide numbering is according to the reference transcripts *TTN* NM_001267550.2; *CHRNG* NM_005199.5; *RYR1* NM_000540.3; *NEB* NM_001164508.2; *COL6A1* NM_001848.3; *ACTA1* NM_001100.4; *LAMA2* NM_000426.4.

Abbreviations: AAF, alternative allelic frequency; ASD, autism spectrum disorder; ID, intellectual disability; LP, likely pathogenic; NIV, non‐invasive ventilation; NMJ, neuromuscular junction; NNIV, nocturnal non‐invasive ventilation; P, pathogenic; TRAC, permanent tracheostomy; VUS, variant of uncertain significance. The patients’ genetic variants are shown in bold.

Most individuals were of Spanish origin (80/105, 76%), followed by Moroccan (13/105, 12%), Latin American (5/105, 5%), and Romanian (3/105, 3%) origins. Additionally, there was one individual each of Emirati, Nigerian, Ukrainian, and Pakistani backgrounds (1/105, 1% each). The mean age at the first clinical evaluation was 3.73 years (SD 3.7), while the mean age at the last evaluation was 12.8 years (SD 7.8), with ages ranging from the neonatal period to 48 years. A genetic diagnosis was established in 55 out of 105 individuals (52%), corresponding to 51 out of 101 families (50%). Among those with a genetic diagnosis, the mean age at diagnosis was 9.47 years (SD 7.9). Four individuals died before the conclusion of this study, all due to respiratory failure during their first year of life, with a median age at death of 3 months (IQR 0.1–0.7). Three of these four individuals had a myogenic profile associated with variants in *NEB* (two individuals) and *ANTXR2* (one individual).

**FIGURE 2:**
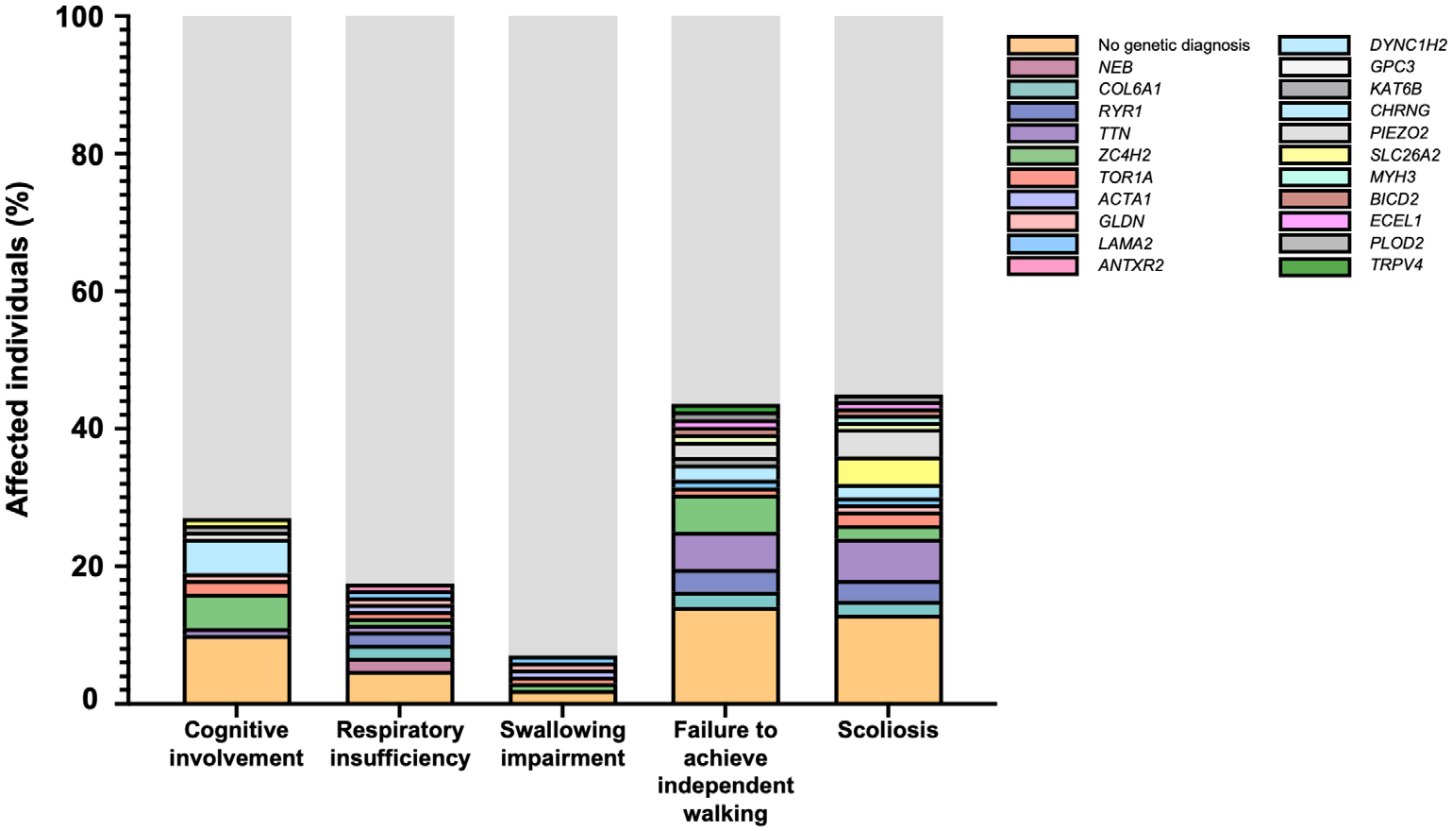
Proportion of all individuals with AMC who had cognitive involvement, respiratory insufficiency, swallowing impairment, failure to achieve independent walking, and scoliosis, along with the distribution of altered genes for each of these characteristics.

Cognitive involvement, including individuals with intellectual disability and/or autism spectrum disorder, was observed in 27% of cases (27/100), mostly associated with pathogenic variants in *DYNC1H1*, *TOR1A*, and *ZC4H2* (Figure 2 and Tables 1, 2, 3). This analysis was performed in individuals older than 2 years, as cognitive assessment in younger children was not considered reliable. Brain MRI scans were available for 10 of the 12 individuals with variants in *DYNC1H1*, *ZC4H2*, or *TOR1A* and revealed abnormalities in all but one case with *DYNC1H1*‐related AMC. Cortical migration defects were the most common findings in *DYNC1H1*‐related cases, whereas white matter volume loss was the predominant abnormality in individuals with *ZC4H2* variants. Among the remaining genes in the cohort, for which at least two individuals were included, brain MRI results were either normal or the individuals did not undergo MRI. Respiratory insufficiency requiring ventilatory support affected 17.1% of individuals (18/105), with a mean age of onset of 4.3 years (SD 4.78, range: newborn–15 years). The causative genes identified in individuals requiring ventilatory support were *COL6A1* (two individuals), *RYR1* (two individuals), and *ACTA1*, *GLDN*, *LAMA2*, *TOR1A*, *TTN*, and *ZC4H2*, each in one individual (Figure 2 and Tables 1 and 2). Additionally, 6.9% of the entire cohort (7/102), including individuals with and without a genetic diagnosis, had swallowing impairment at the last follow‐up and required gastrostomy. All of them also exhibited respiratory involvement and needed ventilatory support. Five individuals with swallowing impairment had a genetic diagnosis and carried pathogenic variants in *ACTA1*, *GLDN*, *LAMA2*, *TOR1A*, and *ZC4H2*, with one case reported for each gene (Figure 2).

**TABLE 2:** Clinical and genetic information of all genetically diagnosed individuals with non‐myogenic AMC of our cohort.

Ind	Fam	Sex	Age last seen/death (year)	Etiological group	Gene (pattern of inheritance)	Variant(s) ACMG evidence of pathogenicity	Age at independent gait (year)	Respiratory Support (age; year)	Scoliosis (Surgery: Yes/No; age at surgery (year))	Cognitive involvement
34	30	F	15	Neurogenic	*DYNC1H1* (AD)	**c.4867C>T; p.Arg1623Trp** (P (PS4, PP2, PP3, PM2, PM5, PM6))	4	No	Yes (surgery at 15)	ID and ASD
35	31	F	11	Neurogenic	*DYNC1H1* (AD)	**c.1741A>T; p.Met581Leu** (P (PS4, PP2, PP3, PM1, PM2, PM6))	1,3	No	No	ID
36	32	M	9	Neurogenic	*DYNC1H1* (AD)	**c.751C>T; p.Arg251Cys** (LP (PS2, PP2, PM2, PM5))	Not achieved	No	No	ID
37	33	F	17	Neurogenic	*DYNC1H1* (AD)	**c.751C>T; p.Arg251Cys** (LP (PS2, PP2, PM2, PM5))	1,6	No	Yes (no surgery)	ID
38	34	M	11	Neurogenic	*DYNC1H1* (AD)	**c.6880G>A; p.Glu2294Lys** (LP (PS4, PM2, PM6, PP2, PP3))	Not achieved	No	No	ID
39	35	F	13	Neurogenic	*ZC4H2* (AD)	c.551delC; p.Pro184Hisfs*3 (LP (PVS1, PM2, PM6))	Not achieved	NNIV (3 year)	Yes (surgery at 14)	ID
40	36	F	3	Neurogenic	*ZC4H2* (AD)	c.199C>T; p.Arg67* (P (PVS1, PS2, PS3, PM2))	Not achieved	No	No	ID
41	37	M	6	Neurogenic	*ZC4H2* (AD)	c.158_171del; p.Asp53Glyfs*15 (P (PVS1, PM2, PM6)) *Variant in mosaic – AAF: 25%*	Not achieved	No	No	ID
42	38	M	4	Neurogenic	*ZC4H2* (AD)	**c.399‐1G>T** (P (PVS1, PM2, PM6))	Not achieved	No	No	ID
43	39	F	13	Neurogenic	*ZC4H2* (AD)	c.34G>T; p.Glu12* (P (PVS1, PM2, PM6))	Not achieved	No	Yes (no surgery)	ID
44	40	M	22	Neurogenic	*BICD2* (AD)	**c.614 T>C; p.Phe205Ser** (VUS (PP3, PM2, PM6))	Not achieved	No	Yes (surgery at 13)	No
45	41	M	3	Neurogenic	*ECEL1* (AR)	**c.2151 + 2 T>C** (P (PVS1, PM2, PM3)) **c.1843A>C; p.Thr615Pro** (LP (PP3, PM2, PM3))	Not achieved	No	Yes (no surgery)	No
46	42	M	7	Neurogenic	*GLDN* (AR)	c.319_325del; p.Glu107Metfs*4 (LP (PVS1, PM2)) c.1435C>T; p.Arg479* (LP (PVS1, PP5, PM2))	2,5	NNIV (< 1 year)	Yes (surgery at 9)	ASD
47	43	F	11	Neurogenic	*TRPV4* (AD)	**c.806G>A; p.Arg269His** (P (PS2, PS3, PP1, PM1, PM2, PM5))	52	48	F	11
48	44	M	14	CNS defects	*TOR1A* (AR)	**c.‐3G>T** (VUS (PM2, BP7)) **c.336C>A; p.Ser112Arg** (VUS (PM2))	3	No	Yes (no surgery)	ID
49	45	F	10	CNS defects	*TOR1A* (AR)	c.862C>T; p.Arg288* (P (PS4, PVS1, PM2)) **c.907_909del; p.Glu303del** (P (PS3, PM2, PM3, PM4))	Not achieved	NNIV (5 year)	Yes (no surgery)	ID
50	46	M	12	CNS defects	*GPC3* (AD)	c.1053G>A; p.Trp351* (VUS (PM2))	3	No	No	ID
51	47	F	5	CNS defects	*KAT6B* (AD)	c.3788_3789delAA; p.Lys1263Argfs*7 (P (PVS1, PS2, PP5, PM2))	Not achieved	No	No	ID
52	48	M	7	Connective tissue and skeletal dysplasia.	*SLC26A2* (AR)	c.532C>T;p.Arg178* (P (PVS1, PS3, PM2, PM3)) **c.1957 T>A;p.Cys653Ser** (P (PS3, PP1, PP3, PM1, PM2, PM3, PM5))	Not achieved	No	Yes (no surgery)	No
53	49	M	11	Connective tissue and skeletal dysplasia.	*SLC26A2* (AR)	**c.835C>T; p.Arg279Trp** (P (PS3, PP3, PM1, PM2, PM3)) **c.835C>T; p.Arg279Trp** (P (PS3, PP3, PM1, PM2, PM3))	1,3	No	No	No
54	50	F	0, 7 (death)	Connective tissue and skeletal dysplasia	*ANTXR2* (AR)	c.903dup; p.Ser302Ilefs*16 (P (PVS1, PM2, PM3)) c.903dup; p.Ser302Ilefs*16 (P (PVS1, PM2, PM3))	< 1 year at last visit	Death due to severe respiratory involvement	< 1 year at last visit	< 1 year at last visit
55	51	M	12	Connective tissue and skeletal dysplasia	*PLOD2* (AR)	**c.1864G>T; p.Gly622Cys** (VUS (PP3, PM1, PM2, PM5)) **c.2122‐2A>G** (LP (PVS1, PP5, PM2))	Not achieved	No	Yes (no surgery)	No

*Note:* All variants are annotated in hg19/GRCh37 and nucleotide numbering is according to the reference transcripts *DYNC1H1* NM_001376.5; *ZC4H2* NM_018684.4; *PIEZO2* NM_001378183.1; *TOR1A* NM_000113.3; *SLC26A2* NM_000112.4; *ANTXR2* NM_058172.6; *BICD2* NM_001003800.2; *ECEL1* NM_004826.4; *PLOD2* NM_182943.3; *TRPV4* NM_021625.5; *GPC3* NM_001164617.2; *KAT6B* NM_012330.4. Variant classification was done based on the American College of Medical Genetics and Genomics (ACMG) guidelines [19], using Franklin (last access November 2024). Evidence of pathogenic and/or benign impact of each variant is indicated.

Abbreviations: AAF, alternative allelic frequency; ASD, autism spectrum disorder; ID, intellectual disability; LP, likely pathogenic; NIV, non‐invasive ventilation; NMJ, neuromuscular junction; NNIV, nocturnal non‐invasive ventilation; P, pathogenic; TRAC, permanent tracheostomy; VUS, variant of uncertain significance. The patients’ genetic variants are shown in bold.

### 3.2 Motor Function and Scoliosis of the Whole Cohort

In our cohort, 55% of individuals (52/94) achieved independent walking, with a mean age of walking onset at 20 months (IQR 11‐60). Nine individuals were not included in this analysis because they were younger than 2 years at the last follow‐up or due to missing data. At the last follow‐up, 96% of those who had acquired ambulation (50/52) retained the ability to walk (Figure 2 and Tables 1, 2, 3). None of the individuals with arthrogryposis caused by pathogenic variants in *COL6A1*, *RYR1*, or *ZC4H2* achieved independent walking. Additionally, there were individuals with AMC and pathogenic variants in other genes (*BICD2*, *DYNC1H1, ECEL1*, *KAT6B*, *LAMA2*, *PIEZO2*, *PLOD2*, *SLC26A2*, *TRPV4*, *and TTN*) who also did not attain the ability to walk.

**TABLE 3:** Clinical information of all individuals with AMC of our cohort without a genetic diagnosis.

Ind	Fam	Sex	Age last seen/death (year)	Etiological group	Age at independent gait (year)	Respiratory support (age; year)	Scoliosis (surgery: Yes/No; age at surgery (year))	Cognitive involvement
56	52	M	15	Maternal illness or exposures	1,4	NNIV (15 year)	Yes (surgery at 15 year)	No
57	53	F	15	Amyoplasia	Not achieved	No	Yes (no surgery)	No
58	54	M	15	Amyoplasia	1,1	NNIV	No	No
59	55	M	3	Amyoplasia	Not achieved	No	No	No
60	56	F	6	Amyoplasia	Not achieved	No	No	No
61	57	F	16	Neurogenic	Not achieved	No	Yes (no surgery)	ID
62	58	M	3	CNS defects	Not available	No	Yes (no surgery)	ID
63	59	F	17	Neurogenic	2	No	No	No
64	60	M	13	Neurogenic	Not achieved	No	Yes (surgery at 9 year)	ID
65	61	M	17	Neurogenic	2	No	Yes (surgery at 8 year)	No
66	62	F	18	Neurogenic	Not available	No	No	No
67	63	F	16	Neurogenic	Not available	No	No	No
68	64	M	18	Neurogenic	1,1	No	Yes (no surgery)	No
69	65	F	16	Not categorized in any specific group	1,1	No	Yes (surgery at 11 year)	No
70	66	F	16	Neurogenic	2	No	No	No
71	67	M	21	Neurogenic	1,2	No	No	No
72	68	F	14	Not categorized in any specific group	1,2	No	No	No
73	69	M	14	Neurogenic	2	No	No	No
74	70	F	12	Not categorized in any specific group	5	NNIV	Yes (surgery at 9 year)	No
75	71	M	13	Neurogenic	1, 5	No	No	No
76	72	M	13	Neurogenic	Not achieved	No	No	ID
77	73	F	13	Neurogenic	1, 2	No	No	ID
78	74	F	12	Not categorized in any specific group	2, 5	No	No	No
79	75	F	16	Neurogenic	Not available	No	No	No
80	76	M	14	Neurogenic	1, 3	No	No	No
81	77	M	10	Neurogenic	Not achieved	NNIV (3 year)	No	ID
82	78	F	15	Myogenic (including NMJ and tension sensing genes)	1, 5	No	No	No
83	79	M	19	Neurogenic	Not available	No	No	No (Dyslexia)
84	80	M	10	Neurogenic	2,8	No	No	ID
85	81	M	15	Neurogenic	Not available	No	No	No
86	82	M	18	Neurogenic	2	No	No	No
87	83	M	12	Neurogenic	Not achieved	No	Yes (no surgery)	No
88	84	M	9	CNS defects	Not achieved	No	No	ID
89	85	F	5	Myogenic (including NMJ and tension sensing genes)	Not achieved	No	Yes (surgery at 6 y)	No
90	86	M	15	Neurogenic	Not available	No	No	No
91	87	M	7	Neurogenic	1, 8	No	No	No
92	88	F	4	Not categorized in any specific group	1, 4	No	No	No (Language delay)
93	89	M	18	Not categorized in any specific group	1	No	No	No
94	90	M	0, 1	Not categorized in any specific group	< 1 year at last visit	Death due to severe respiratory involvement	< 1 year at last visit	< 1 year at last visit
95	91	F	1, 6	Myogenic (including NMJ and tension sensing genes)	2, 6	No	No	ID
96	92	F	3	Myogenic (including NMJ and tension sensing genes)	Not achieved	No	No	No
97	93	F	14	Peripheral nerve defects	1, 3	No	No	No
98	94	M	13	Myogenic (including NMJ and tension sensing genes)	1, 3	No	No	No
99	95	F	5	Neurogenic	1, 5	No	No	No
100	96	F	3	Neurogenic	Not achieved	No	Yes (no surgery)	No
101	97	F	2	Not categorized in any specific group	1	No	No	No
102	98	M	13	CNS defects	Not achieved	No	No	ID
103	99	M	5	Neurogenic	1, 3	No	Yes (no surgery)	No
104	100	M	9	Neurogenic	1	No	No	No
105	101	M	48	Myogenic (including NMJ and tension sensing genes)	Not available	No	No	No

Abbreviations: ID, intellectual disability; NNIV, nocturnal non‐invasive ventilation.

Scoliosis was noted in 45/100 individuals, with 17 (38%) undergoing corrective surgery at a mean age of 11.6 years (SD 4.00; range 3–19). Individuals younger than 2 years at the last follow‐up were not included in the scoliosis analysis. A high proportion of individuals requiring corrective scoliosis surgery was identified among those with AMC associated with the following genes, all associated with neuromuscular diseases: *COL6A1* (100%, 2/2), *TOR1A* (100%, 2/2), *RYR1* (100%, 3/3), *CHRNG* (80%, 4/5), *PIEZO2* (80%, 4/5), *TTN* (67%, 6/9), *ZC4H2* (40%, 2/5), and *DYNC1H1* (40%, 2/5) (Figure 2 and Tables 1, 2, 3).

### 3.3 Etiological Stratification

**FIGURE 3:**
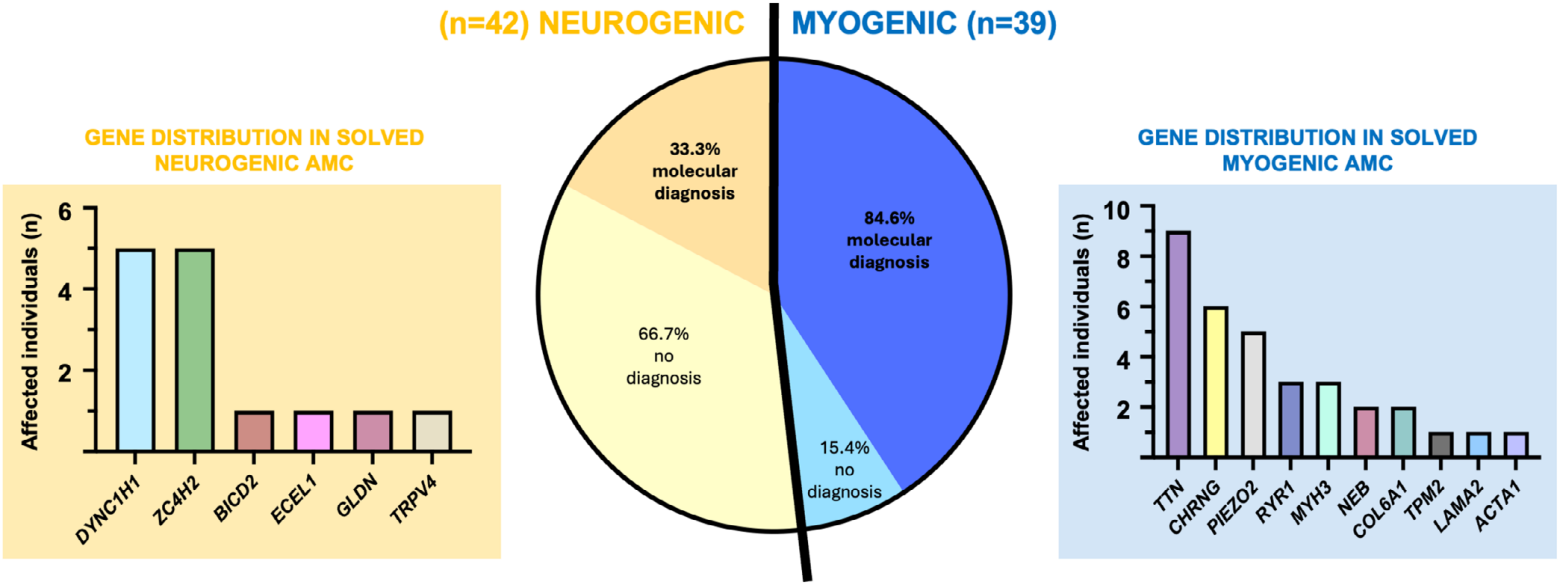
Comparison of the molecular diagnostic rate in individuals with AMC of myogenic or neurogenic cause, with gene distribution specified for each category. Genetic diagnosis was achieved in 84.6% of individuals with myogenic AMC, while it was reached in only 33.3% of individuals with neurogenic AMC.

Within the total cohort, 81 individuals (77.1%) had arthrogryposis of neuromuscular origin, with defects involving motor neurons/peripheral nerves (42 individuals, 40%), the neuromuscular junction (6 individuals, 5.7%), and skeletal muscle (33 individuals, 31.4%) (Figure 3 and Tables 1, 2, 3). Seven individuals (6.7%) were classified under the CNS subgroup, while four individuals (3.8%) were classified into the connective tissue and Amyoplasia groups. One individual (1%) with fetal acetylcholine receptor inactivation syndrome was categorized under the maternal illness or exposures group, and eight individuals (7.6%) could not be classified.

### 3.4 Gene Distribution and Genotype–Phenotype Correlations

**FIGURE 4:**
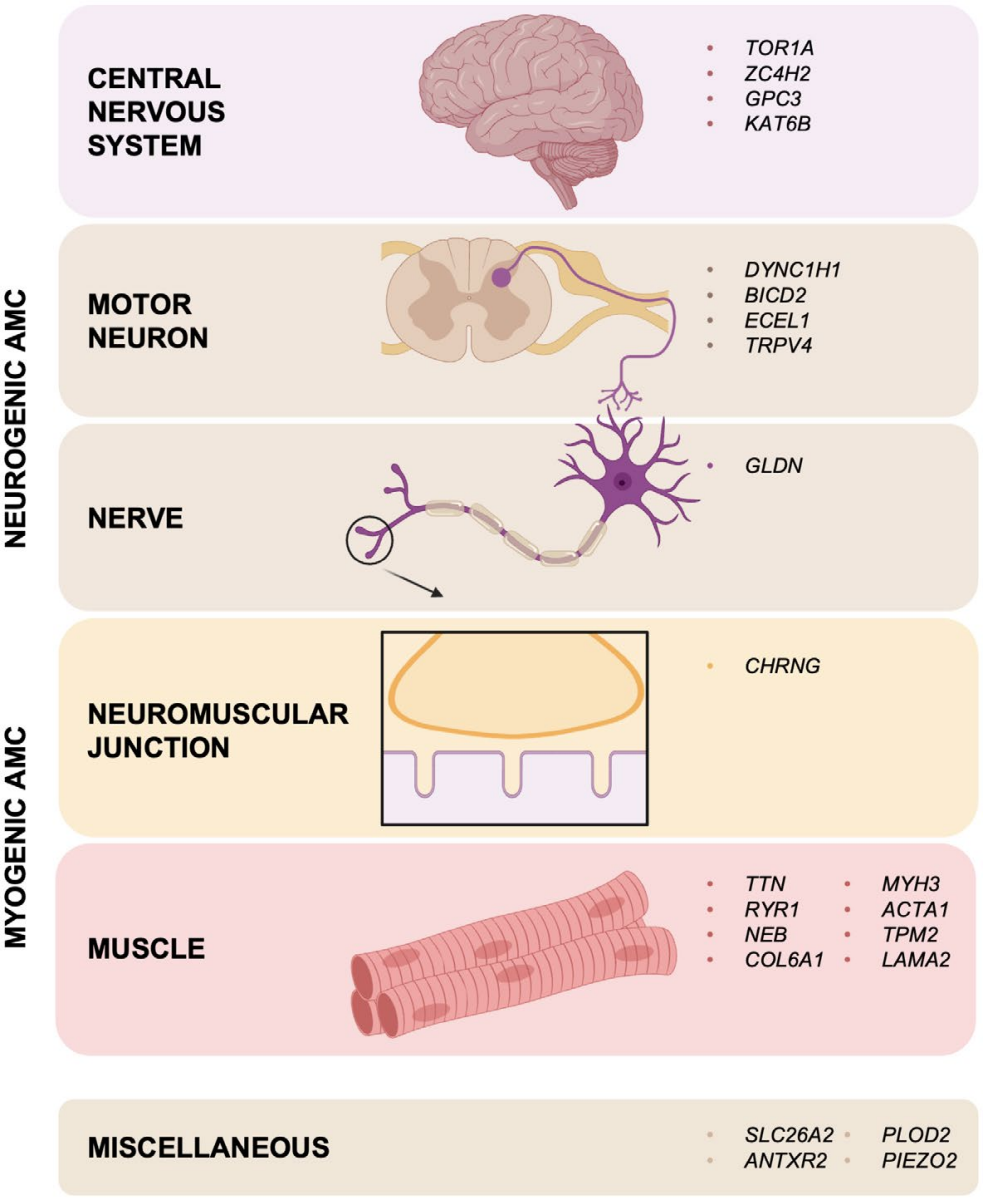
Schematic summary of genes causing AMC identified in this cohort. Gene products have been classified according to their main functions.

A genetic diagnosis was achieved in 55 of 105 individuals (52%), corresponding to 51 of 100 families (51%) (Tables 1 and 2). The most frequently identified gene was *TTN*, accounting for 16% of the genetically resolved cases (9 of 55). Other common genes included *CHRNG* (6/55; 10.9%), *PIEZO2* (5/55; 9.1%), *ZC4H2* (5/55; 9.1%), *DYNC1H1* (4/55; 7.3%), *MYH3* (3/55; 5.4%), and *RYR1* (3/55; 5.4%). Figure 1 illustrates the distribution of affected individuals according to the specific etiology of AMC, while Figure 4 presents a schematic representation of the genes identified in this cohort. No cases due to congenital myotonic dystrophy or spinal muscular atrophy type 0 were identified.

A total of 65 unique genetic variants were identified across 22 different genes. Tables 1 and 2 show the causative variants and their classification according to ACMG guidelines [10].

The diagnostic rate among the 81 individuals with arthrogryposis of neuromuscular origin varied significantly (Figure 3). The rate was notably lower for individuals with neurogenic AMC (14 out of 42 individuals, 33.3%) compared with those with myogenic AMC (33 out of 39 individuals, 84.6%). Gene panel testing yielded a diagnosis in 32% of cases (13/40), while exome sequencing had a slightly higher diagnostic rate of 36% (15/42). Trio‐based genome sequencing was performed in 23 individuals who remained undiagnosed after panel testing, achieving a diagnostic yield of 39% (9/23).

The most frequently altered genes among individuals with neurogenic AMC were *ZC4H2* and *DYNC1H1*, accounting for five and four individuals, respectively (5/42; 11.9% and 4/42; 9.5% of all genetically explained neurogenic AMC cases) (Figure 3). These nine individuals exhibited a phenotype characterized by intellectual disability associated with motor axonal neuropathy, identifiable through ENMG. Other genes identified in neurogenic AMC cases included *BICD2*, *ECEL1*, *GLDN*, and *TRPV4*, with one case reported for each gene (1/42; 2.4%).

Among individuals with myogenic AMC, the most frequently affected gene was *TTN* (9/39 individuals, 23.1%), followed by *RYR1* and *MYH3* (3/39 individuals each, 7.7%), and *NEB* and *COL6A1* (2/39 individuals each, 5.1%) (Figure 3). Variants in *LAMA2* and *ACTA1* were found in one individual each (1/39, 2.6%). In 6/39 individuals (15.4%) the altered gene was *CHRNG*, and in 5/39 (12.8%) had altered *PIEZO2*. These individuals were included in the myogenic AMC group despite *CHRNG* and *PIEZO2* not being strictly muscle‐specific genes, as they typically present myopathic findings on electromyography.

The ability to achieve independent ambulation varied notably across genetic subgroups. None of the individuals with AMC caused by *ZC4H2* or *RYR1* variants acquired independent walking, whereas all six individuals with *CHRNG*‐related AMC achieved independent ambulation. Among individuals with *TTN* variants, 55.6% (5/9) never walked, while the remaining four individuals achieved ambulation at an average age of 2.9 years (range: 1.5–4 years). For *DYNC1H1* variants, half of the individuals (2/4) did not acquire ambulation, whereas the others walked at an average age of 1.5 years.

The prevalence of cognitive impairment also varied across genetic subgroups. Intellectual disability was universally present in individuals with variants in *ZC4H2* (5/5), *DYNC1H1* (4/4), *TOR1A* (2/2), *KAT6B* (1/1), *GLDN* (1/1), and *GPC3* (1/1), signifying a strong association between these genes and cognitive deficits. In contrast, cognitive impairment was observed in only one additional case, an individual with AMC due to pathogenic variants in *TTN*.

A noteworthy observation is the high prevalence of myogenic AMC associated with *TTN* variants. In all such cases, at least one variant was identified within metatranscript‐only regions. These sequences, not included in the skeletal muscle N2A isoform (NM_133379.5), exhibit elevated expression during the prenatal period and have consequently been associated with AMC [11].

### 3.5 Characteristics of Individuals Who Remain Without a Genetic Diagnosis

Despite genetic studies, 44.6% of individuals (45 out of 100, after excluding four individuals with Amyoplasia, in which the cause is assumed to be non‐genetic [9], and one individual with FARAD, where the cause is immunological due to maternal‐origin antibodies [12]) in the entire cohort remain genetically undiagnosed (Table 3). The mean age at the last evaluation for this subgroup was 12.6 years (SD 8.1), which was not significantly different from the mean age of individuals with a genetic diagnosis (13.2 years; SD 7.7).

Clinical exome sequencing or whole exome sequencing was conducted in 41 of the 45 undiagnosed individuals, and additionally, trio genome sequencing was performed in 13 of these individuals. In the remaining four undiagnosed individuals, a limited number of genes were studied individually, and CGH‐array was performed in two cases.

Among individuals without a genetic diagnosis, the proportion who never achieved independent walking was significantly lower (29% vs. 53%). One individual in the undiagnosed subgroup died during their first year of life due to respiratory failure.

In terms of AMC etiology, the majority of undiagnosed cases were neurogenic (28 out of 45 individuals; 62.2%), followed by cases with unclear classification (8/45; 17.8%), myogenic AMC (6/45; 13.3%), and AMC with CNS involvement (3/45; 6.7%). The proportion of neurogenic AMC in the undiagnosed group was notably higher than in the overall cohort, likely due to the lower diagnostic yield in the neurogenic subgroup.

## 4 Discussion

The findings in this study provide a detailed overview of the genetic and phenotypic characteristics in individuals with AMC of neuromuscular origin, contributing to our understanding of fetal akinesia/hypokinesia disorders. The etiological classification established in this study highlights the diversity of genetic factors, with peripheral nerve defects and skeletal muscle abnormalities representing the most prevalent groups. Notably, the myogenic subgroup demonstrated a significantly higher rate of genetic diagnosis than the neurogenic subgroup. This difference in genetic resolution rates underscores the potential for more precise diagnostic algorithms targeting the etiologic subgroup, which could improve diagnostic yield and allow for more individualized clinical management.

The observation of specific genotype–phenotype correlations, such as the increased association of intellectual disability with variants in *DYNC1H1*, *ZC4H2*, and *TOR1A*, is valuable for early prognosis and family counseling. Furthermore, identifying that respiratory insufficiency and nutritional support were associated with a subset of these genes provides a clearer understanding of how these complications are distributed within the genetic subtypes of AMC. Future studies could examine larger cohorts to validate these associations, potentially enabling genotype‐based risk stratification in clinical practice.

The study also points out the limitations of current genetic diagnostic techniques. Despite using advanced NGS approaches, approximately half of the individuals remained genetically unresolved, particularly in neurogenic AMC cases. Although the diagnostic yields of gene panels, exome sequencing, and genome sequencing ranged from 32% to 39%, a direct comparison between these methods is not appropriate due to differences in how they were applied. Gene panels and exome sequencing were primarily used as first‐line tests, whereas genome sequencing was performed only as a second‐tier investigation in individuals who remained undiagnosed after initial testing. Additionally, genome sequencing was mostly performed in a trio format, while exome sequencing was typically conducted in singleton cases.

The diagnostic rate in our cohort is comparable to that observed in other recently published cohorts, which range from 42.6% (81/190) in Ravescroft et al. [3], 52.7% (166/315) in Laquerriere et al. [5], to 66% (83/125) in Le Tanno et al. [4]. However, given the heterogeneity of these cohorts, which include individuals with varying characteristics such as a higher or lower proportion of cases with Amyoplasia or with neurogenic or myogenic etiologies, a direct comparison of the diagnostic strategies employed is not feasible.

Several factors may contribute to the high proportion of unresolved cases in AMC, including the involvement of novel genes (with a suspicion particularly directed at genes with isoforms that are exclusively highly expressed during the fetal period) or the presence of pathogenic variants in regulatory regions that are poorly covered by exome sequencing and remain challenging to interpret even with genome sequencing. Continued efforts to integrate genome sequencing, transcriptomic, and epigenomic analyses may increase the diagnostic yield in these unresolved cases, as well as the periodic reanalysis of sequencing data to identify variants in genes not yet associated with AMC at the time of the initial analysis. Additionally, expanding biobanking and registries of individuals with AMC could facilitate future research into these undiagnosed cases and foster national and international collaborations that are necessary to identify rarer or complex etiologies.

Despite the comprehensive approach in this study, several limitations should be considered. First, its retrospective design relies on clinical data from a single‐center cohort, which may limit the generalizability of the findings to broader populations or different healthcare settings. Additionally, not all individuals underwent systematic neuropsychological assessments, which could have provided a more detailed understanding of the cognitive impact of different genetic variants. Another limitation is that the extent of genetic testing varied among cases, leaving room for further analyses that may help resolve currently unsolved cases. Finally, the lack of long‐term follow‐up data for all individuals may have limited insights into disease progression.

Our study does not aim to determine the true prevalence of each cause of AMC, as this would require a prospective study starting in the gestational period, involving obstetricians and neonatologists, and including pregnancy terminations. In our cohort, focused on individuals with AMC of likely neuromuscular origin, it is understandable that cases with Amyoplasia and maternal causes are underrepresented, likely due to a selection bias, as they may not be referred to our neuromuscular unit.

Finally, this study underscores the importance of phenotypic characterization as an essential complement to genetic analysis. The clinical stratification of individuals with AMC, particularly by neurogenic versus myogenic origins, was instrumental in interpreting the genotypic data and led to a more precise identification of the affected molecular pathways.

## Author Contributions

Florencia Pérez‐Vidarte, Berta Estévez‐Arias, Andres Nascimento, and Daniel Natera‐de Benito conceptualized and designed the study, coordinated and supervised data collection, carried out the initial analyses, drafted the initial manuscript, and critically reviewed and revised the manuscript. Carlos Ortez, Julita Medina, Lidia DeSena deCabo, Laura Carrera‐García, and Jesica Expósito‐Escudero collected clinical data and critically reviewed and revised the manuscript. Anna Codina and Cristina Jou supervised data collection of muscle biopsies and critically reviewed and revised the manuscript. Berta Estévez‐Arias, Leslie Matalonga, Delia Yubero, and Eduardo F. Tizzano supervised data collection of genetics, carried out some analyses, and critically reviewed and revised the manuscript. All authors approved the final manuscript as submitted and agree to be accountable for all aspects of the work.

## Ethics Statement

Data were collected and analyzed following the ethics guidelines of Hospital Sant Joan de Déu (PIC 147‐23). We confirm that we have read the Journal's position on issues involved in ethical publication and affirm that this report is consistent with those guidelines.

## Consent

The families provided written informed consent for the study.

## Conflicts of Interest

The authors declare no conflicts of interest.

## Data Availability

Any data not published within the article will be shared from the corresponding author, upon reasonable request.
